# 1053. The β-Lactamase Inhibitor QPX7728 Restores the Activity of β-Lactam Agents against Contemporary Extended-Spectrum β-lactamase (ESBL)-Producing and Carbapenem-Resistant *Enterobacterales* (CRE) Isolates, Including Isolates Producing Metallo-β-lactamases

**DOI:** 10.1093/ofid/ofab466.1247

**Published:** 2021-12-04

**Authors:** Jill Lindley, Yahse Edah, Olga Lomovskaya, Mariana Castanheira, Mariana Castanheira

**Affiliations:** 1 JMI Laboratories, North Liberty, Iowa; 2 Qpex Biopharma, San Diego, California

## Abstract

**Background:**

The β-lactam (BL)/ β-lactamase inhibitor (BLI) combinations approved in the last 10 years are active against most ESBL-producing *Enterobacterales* (ENT) and CRE isolates, but have limited activity against metallo-β-lactamase (MBL)-producing ENT. We evaluated the activity of QPX7728 (QPX), a novel BLI with intravenous (IV) and oral availability, in combination with BL agents. We tested ENT isolates carrying the most common BL genes such as *bla*_CTX-M_, transferable AmpCs, oxacillinases, MBLs, and serine carbapenemases.

**Methods:**

A total of 1,027 ENT isolates were susceptibility (S) tested by reference broth microdilution against aztreonam (ATM), cefepime (FEP), cefdinir (CDR), ceftibuten (CTB), ceftolozane (CT) and piperacillin (PT) with fixed 4 mg/L of tazobactam, biapenem (BPM), meropenem (MER), and tebipenem (TEB) combined with QPX at fixed 4 and 8 mg/L. All isolates were genetically characterized using whole genome sequencing and included 520 ESBL-producers and 507 CRE with 168 producing MBLs.

**Results:**

BL agents tested alone had limited activity against this challenge set of isolates (MIC_90_, ≥32 mg/L); however, MIC_90_ values decreased ≥32-fold with the addition of QPX at the highest concentration tested (Table). Oral agents, CTB,CDR and TEB were tested with QPX at a fixed 4 mg/L and showed a 32- to 128-fold increase in potency (MIC_90_, 0.5-4 mg/L). ATM and FEP were tested with QPX at a fixed 4 and 8 mg/L and displayed MIC_90_ values ranging from 0.12-0.5 mg/L. ATM and FEP, tested with 8 mg/L of QPX, inhibited 99.8% of isolates at the breakpoint for the BL agent alone. BLI inhibitor combinations PT and CT displayed MIC_90_ values of 2 and 4 mg/L with the addition of 8 mg/L QPX. MER with QPX at a fixed 4 mg/L and 8 mg/L inhibited 99.8% and 100% of isolates, respectively.

**Conclusion:**

The activity of all BLs evaluated was restored when combined with QPX tested against this challenging collection of 1,027 ENT isolates displaying various resistance mechanisms, including difficult to treat CRE isolates and MBL producers. Further development of QPX with various orally- and IV-available BL agents appears warranted.

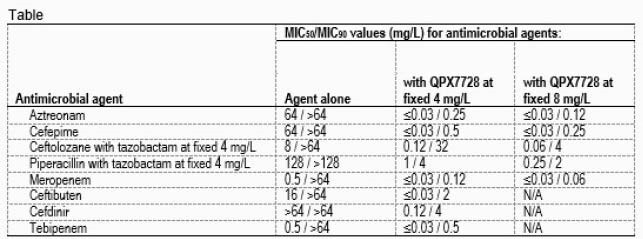

**Disclosures:**

**Jill Lindley**, **Bravos Biosciences** (Research Grant or Support)**ContraFect Corporation** (Research Grant or Support)**Pfizer, Inc.** (Research Grant or Support)**Qpex Biopharma** (Research Grant or Support) **Yahse Edah, AS**, **Qpex** (Research Grant or Support) **Olga Lomovskaya, PhD**, **Qpex Biopharma** (Employee) **Mariana Castanheira, PhD**, **AbbVie (formerly Allergan**) (Research Grant or Support)**Bravos Biosciences** (Research Grant or Support)**Cidara Therapeutics, Inc.** (Research Grant or Support)**Cipla Therapeutics** (Research Grant or Support)**Cipla USA Inc.** (Research Grant or Support)**GlaxoSmithKline** (Research Grant or Support)**Melinta Therapeutics, Inc.** (Research Grant or Support)**Melinta Therapeutics, LLC** (Research Grant or Support)**Pfizer, Inc.** (Research Grant or Support)**Qpex Biopharma** (Research Grant or Support)**Shionogi** (Research Grant or Support)**Spero Therapeutics** (Research Grant or Support) **Mariana Castanheira, PhD**, Affinity Biosensors (Individual(s) Involved: Self): Research Grant or Support; Allergan (Individual(s) Involved: Self): Research Grant or Support; Amicrobe, Inc (Individual(s) Involved: Self): Research Grant or Support; Amplyx Pharma (Individual(s) Involved: Self): Research Grant or Support; Artugen Therapeutics USA, Inc. (Individual(s) Involved: Self): Research Grant or Support; Astellas (Individual(s) Involved: Self): Research Grant or Support; Basilea (Individual(s) Involved: Self): Research Grant or Support; Beth Israel Deaconess Medical Center (Individual(s) Involved: Self): Research Grant or Support; BIDMC (Individual(s) Involved: Self): Research Grant or Support; bioMerieux Inc. (Individual(s) Involved: Self): Research Grant or Support; BioVersys Ag (Individual(s) Involved: Self): Research Grant or Support; Bugworks (Individual(s) Involved: Self): Research Grant or Support; Cidara (Individual(s) Involved: Self): Research Grant or Support; Cipla (Individual(s) Involved: Self): Research Grant or Support; Contrafect (Individual(s) Involved: Self): Research Grant or Support; Cormedix (Individual(s) Involved: Self): Research Grant or Support; Crestone, Inc. (Individual(s) Involved: Self): Research Grant or Support; Curza (Individual(s) Involved: Self): Research Grant or Support; CXC7 (Individual(s) Involved: Self): Research Grant or Support; Entasis (Individual(s) Involved: Self): Research Grant or Support; Fedora Pharmaceutical (Individual(s) Involved: Self): Research Grant or Support; Fimbrion Therapeutics (Individual(s) Involved: Self): Research Grant or Support; Fox Chase (Individual(s) Involved: Self): Research Grant or Support; GlaxoSmithKline (Individual(s) Involved: Self): Research Grant or Support; Guardian Therapeutics (Individual(s) Involved: Self): Research Grant or Support; Hardy Diagnostics (Individual(s) Involved: Self): Research Grant or Support; IHMA (Individual(s) Involved: Self): Research Grant or Support; Janssen Research & Development (Individual(s) Involved: Self): Research Grant or Support; Johnson & Johnson (Individual(s) Involved: Self): Research Grant or Support; Kaleido Biosceinces (Individual(s) Involved: Self): Research Grant or Support; KBP Biosciences (Individual(s) Involved: Self): Research Grant or Support; Luminex (Individual(s) Involved: Self): Research Grant or Support; Matrivax (Individual(s) Involved: Self): Research Grant or Support; Mayo Clinic (Individual(s) Involved: Self): Research Grant or Support; Medpace (Individual(s) Involved: Self): Research Grant or Support; Meiji Seika Pharma Co., Ltd. (Individual(s) Involved: Self): Research Grant or Support; Melinta (Individual(s) Involved: Self): Research Grant or Support; Menarini (Individual(s) Involved: Self): Research Grant or Support; Merck (Individual(s) Involved: Self): Research Grant or Support; Meridian Bioscience Inc. (Individual(s) Involved: Self): Research Grant or Support; Micromyx (Individual(s) Involved: Self): Research Grant or Support; MicuRx (Individual(s) Involved: Self): Research Grant or Support; N8 Medical (Individual(s) Involved: Self): Research Grant or Support; Nabriva (Individual(s) Involved: Self): Research Grant or Support; National Institutes of Health (Individual(s) Involved: Self): Research Grant or Support; National University of Singapore (Individual(s) Involved: Self): Research Grant or Support; North Bristol NHS Trust (Individual(s) Involved: Self): Research Grant or Support; Novome Biotechnologies (Individual(s) Involved: Self): Research Grant or Support; Paratek (Individual(s) Involved: Self): Research Grant or Support; Pfizer (Individual(s) Involved: Self): Research Grant or Support; Prokaryotics Inc. (Individual(s) Involved: Self): Research Grant or Support; QPEX Biopharma (Individual(s) Involved: Self): Research Grant or Support; Rhode Island Hospital (Individual(s) Involved: Self): Research Grant or Support; RIHML (Individual(s) Involved: Self): Research Grant or Support; Roche (Individual(s) Involved: Self): Research Grant or Support; Roivant (Individual(s) Involved: Self): Research Grant or Support; Salvat (Individual(s) Involved: Self): Research Grant or Support; Scynexis (Individual(s) Involved: Self): Research Grant or Support; SeLux Diagnostics (Individual(s) Involved: Self): Research Grant or Support; Shionogi (Individual(s) Involved: Self): Research Grant or Support; Specific Diagnostics (Individual(s) Involved: Self): Research Grant or Support; Spero (Individual(s) Involved: Self): Research Grant or Support; SuperTrans Medical LT (Individual(s) Involved: Self): Research Grant or Support; T2 Biosystems (Individual(s) Involved: Self): Research Grant or Support; The University of Queensland (Individual(s) Involved: Self): Research Grant or Support; Thermo Fisher Scientific (Individual(s) Involved: Self): Research Grant or Support; Tufts Medical Center (Individual(s) Involved: Self): Research Grant or Support; Universite de Sherbrooke (Individual(s) Involved: Self): Research Grant or Support; University of Iowa (Individual(s) Involved: Self): Research Grant or Support; University of Iowa Hospitals and Clinics (Individual(s) Involved: Self): Research Grant or Support; University of Wisconsin (Individual(s) Involved: Self): Research Grant or Support; UNT System College of Pharmacy (Individual(s) Involved: Self): Research Grant or Support; URMC (Individual(s) Involved: Self): Research Grant or Support; UT Southwestern (Individual(s) Involved: Self): Research Grant or Support; VenatoRx (Individual(s) Involved: Self): Research Grant or Support; Viosera Therapeutics (Individual(s) Involved: Self): Research Grant or Support; Wayne State University (Individual(s) Involved: Self): Research Grant or Support

